# Cost-effectiveness of enzyme replacement therapy with alglucosidase alfa in adult patients with Pompe disease

**DOI:** 10.1186/s13023-017-0731-0

**Published:** 2017-12-13

**Authors:** Tim A. Kanters, Ans T. van der Ploeg, Michelle E. Kruijshaar, Dimitris Rizopoulos, W. Ken Redekop, Maureen P. M. H. Rutten-van Mӧlken, Leona Hakkaart-van Roijen

**Affiliations:** 1 Institute for Medical Technology Assessment, Erasmus University Rotterdam, PO Box 1738, 3000DR Rotterdam, the Netherlands; 2 Center for Lysosomal and Metabolic Diseases, Department of Pediatrics Sophia’s Children’s Hospital, Rotterdam, the Netherlands; 3 Department of Biostatistics, Erasmus University Medical Center, Rotterdam, the Netherlands; 4 Erasmus School of Health Policy & Management, Erasmus University Rotterdam, Rotterdam, the Netherlands

**Keywords:** Pompe disease, Enzyme replacement therapy, Orphan drug, Cost-effectiveness, QALY

## Abstract

**Background:**

Pompe disease is a rare, progressive, metabolic disease, and the first treatable inheritable muscle disorder. Enzyme replacement therapy (ERT) with alglucosidase alfa is disease specific and the only medicinal product authorized for the treatment of Pompe disease. Costs of ERT are very high as for most orphan drugs. This study investigates the cost-effectiveness of ERT compared to supportive treatment in adult patients with Pompe disease.

**Methods:**

Survival probabilities were estimated from an international observational dataset (*n* = 283) using a time-dependent Cox model. Quality of life was estimated on a Dutch observational dataset using a previously developed conceptual model which links clinical factors to quality of life. Costs included costs of ERT, costs of drug administration and other healthcare costs. Cost-effectiveness was estimated using a patient-level simulation model (*n* = 90), synthesising the information from underlying models for survival, quality of life and costs. The cost-effectiveness model estimated the (difference in) lifetime effects and costs for both treatments. Two scenarios were modelled: (1) a worse case scenario with no extrapolation of the survival gain due to ERT beyond the observed period (i.e. from 10 years onwards); and (2) a best case scenario with lifetime extrapolation of the survival gain due to ERT. Effects were expressed in (quality adjusted) life years (QALYs). Costs were discounted at 4.0% and effects at 1.5%.

**Results:**

Substantial increases in survival were estimated – discounted incremental life years of ERT ranged from 1.9 years to 5.4 years in the scenarios without and with extrapolation of survival gains beyond the observed period. Quality of life was also significantly better for patients receiving ERT. Incremental costs were considerable and primarily consisted of the costs of ERT. Incremental costs per QALY were €3.2 million for scenario 1 and €1.8 million for scenario 2.

**Conclusions:**

The availability of extended, prospectively collected, longitudinal observational data on the most important input parameters required to construct a cost-effectiveness model is quite exceptional for orphan diseases. The cost-effectiveness model showed substantial survival gains from ERT. Despite these substantial gains, ERT was not cost-effective in the treatment of adult Pompe disease because of the high cost of treatment.

**Electronic supplementary material:**

The online version of this article (10.1186/s13023-017-0731-0) contains supplementary material, which is available to authorized users.

## Background

Pompe disease (or glycogenosis type II) is a rare inheritable muscle disease that also belongs to the glycogen and lysosomal storage disorders. It is caused by a deficiency of the enzyme acid α-glucosidase [1]. The disease has a continuous clinical spectrum of phenotypes, with the progressive, classic-infantile form at the severe end of the spectrum and the late-onset form at the least severe end. In adult patients, the disease particularly affects skeletal muscle and respiratory function, and patients eventually become wheelchair bound and ventilator dependent [1–3]. The frequency of adult Pompe disease is estimated to be 1 per 57:000 people [4]. Compared to the general population, adults with Pompe disease experience a reduced life expectancy and quality of life [5–7].

Enzyme replacement therapy (ERT) with alglucosidase alfa (Myozyme®, Genzyme corp.) has been developed as a disease specific treatment for Pompe disease. Before ERT became available in 2006, patients received supportive treatment (ST) only, consisting of respiratory support, ambulatory support, physiotherapy and/or dietary treatment [8]. In adult patients with Pompe disease, ERT has been shown to improve muscle strength, respiratory function and quality of life [9, 10]. Furthermore, it leads to a significant improvement in survival in both infants and adults [11–13]. Like other treatments for rare diseases, ERT is very expensive. Costs-effectiveness is one of the criteria on which reimbursement decisions are based. In cost-effectiveness studies, the ratio of incremental costs and incremental effects of a new treatment versus a comparator is calculated. In this study, we examined, for the Dutch situation, the cost-effectiveness of ERT compared to ST in adult patients with Pompe disease. Considering the costs associated with ERT, the treatment is not expected to have a favourable cost-effectiveness ratio. Still, it is necessary to conduct economic evaluations, as it provides policy makers with an instrument to engage in price negotiations with drug manufacturers and a comparison of the cost-effectiveness of treatments with other orphan drugs can contribute to the debate on whether or not, for orphan drugs, we may need reimbursement decisions that explicitly incorporate broader societal preferences.

## Methods

The Dutch health economic guidelines were followed in this cost-effectiveness study. A patient-level simulation model was developed to assess cost-effectiveness of ERT for adult patients (i.e. 18 years or older) with Pompe disease. In such a model, outcomes are calculated for individual patients and then the average is taken over the total patient population included [14]. The model compared two treatments: supportive treatment (ST) and enzyme replacement therapy with supportive treatment (ERT). The model was composed of three main components, i.e. survival, quality of life, and costs, which were modelled on the basis of an individual patient’s characteristics for both treatments.

### Survival

Survival probabilities were derived from an international dataset with observational data of patients with Pompe disease (the International Pompe Association (IPA)/Erasmus MC Pompe Survey; *n* = 283), which started to collect data in 2002, 4 years before ERT received market authorization. This database was previously used to estimate survival of adult patients with and without ERT by means of a time-dependent Cox regression model using wheelchair use, ventilator support and treatment as predictors [13]. For the cost-effectiveness model, this survival model was adapted to estimate the baseline hazard for both treatments using the same dataset. This method provides a life table for both treatments (estimated cumulative survival probabilities are provided in Table S1 (see Additional file 1). To make optimal use of all available data, patients contribute data to the survival estimates of both treatments; i.e. patients that received ERT also contributed data to estimate the survival in the ST group before they received ERT. For both treatments, the mean observed follow-up was approximately 3.5 years with a maximum total follow-up for ST of 8.9 years and for ERT of 8.4 years.

Because the survival after the observed period is uncertain, two scenarios were modelled. In scenario 1, a conservative approach was used, assuming no effects of ERT on survival after the observed period. Hence, from year 10 onwards, the survival probabilities estimated for ST at 9 years were kept constant and applied to both treatments in this scenario. This scenario presents a worse case scenario, as no further improvements in survival due to ERT were assumed beyond the observed survival gains, resulting in the lower boundary of survival gains due to ERT. In scenario 2, the effect of ERT on survival was extrapolated beyond the observation period, by carrying forward the estimated treatment-specific survival probabilities of year nine (see Additional file 1: Table S1) for both treatments. To adjust for an increasing risk of mortality with increasing age, the estimated probabilities were replaced by age-based mortality rates for the general Dutch population when these were larger than disease-specific mortality rates. Mortality rates for the Dutch population were derived from Statistics Netherlands [15].

### Quality of life

A previously developed conceptual model for adult Pompe disease, connecting clinical parameters with quality of life [16] was used to obtain estimates for an individual patient’s quality of life. The conceptual model describes the relations between enzyme activity, muscle strength, respiratory function, fatigue, level of handicap, general health perceptions, and utility. The quality of life model in the cost-effectiveness model resembled the conceptual model for adult Pompe disease, except that the estimates from the conceptual model were recalculated using a model specification that included ERT as a covariate, in order to model treatment effects. The other covariates in the quality of life model were age, disease duration and enzyme activity. Using the regression estimates from the conceptual model in combination with patient characteristics (age, disease duration, enzyme activity and treatment), the patient’s muscle strength and respiratory function were estimated. The estimated values for muscle strength and respiratory function were used as input values (next to the patient characteristics) in the regression model for the subsequent level in the conceptual model, i.e. fatigue. Fatigue was used, in combination with muscle strength, respiratory function and patient characteristics, to estimate the next level in the conceptual model, namely handicap level. Handicap and patient characteristics were used in turn to estimate health perceptions. The final level in the conceptual model, i.e. quality of life, was estimated on the basis of the patient’s estimated health perceptions, and the patient characteristics (age, disease duration, enzyme activity and treatment). Regression estimates for the quality of life model were based on a Dutch dataset (*n* = 82), consisting of a sample of all Dutch patients being monitored (both treated and untreated) by the national reference center for Pompe disease (Erasmus MC, University Medical Center, Rotterdam, the Netherlands). Regression estimates are provided in Table S2 (see Additional file 1). The ERT covariate in the quality of life model showed a significant positive effect on utility: utilities for ERT were 0.028 points higher than for ST (*p* = 0.008, (see Additional file 1: Table S2)).

Quality of life was expressed in utilities, which represents the value of a patients’ quality of life on a scale anchored at 1 (perfect health) and 0 (death). Utilities were derived from the EQ-5D questionnaire [17]. Dutch tariffs were used to calculate utilities [18].

### Costs

Costs were calculated from the societal perspective and expressed in 2014 euros. Several costs components were included in the cost-effectiveness model.

ERT costs were based on patients’ weights; dosage was 20 mg/kg body weight every 2 weeks. Patients’ weights were estimated using a random effects model, including age and gender as explanatory variables. The estimates of bodyweight were based on a dataset from the hospital pharmacy on the Dutch patients being treated with ERT at Erasmus MC, University Medical Center, Rotterdam, the Netherlands (*n* = 84). Patients’ weights were multiplied by the list price of medication – ERT costs per kilogram bodyweight were €5788 per year. Because of organizational efficiency in the hospital pharmacy, spillage was very low; therefore, no costs of spillage were included.

Cost of drug administration were based on biweekly infusions. Infusions were provided outside the hospital (mostly at home) for 79% of patients. Based on bottom-up costing research, these costs of drug administration outside the hospital were estimated to be €433; compared to €507 for in-hospital drug administration. A weighted average was used to calculate costs of drug administration.

Other healthcare costs were retrieved from health economic questionnaires [6] among Dutch patients (*n* = 87) at Erasmus MC, University Medical Center, Rotterdam, the Netherlands. These costs related to hospitalizations, outpatient visits, GP visits, paramedical care, home care, diagnostic procedures and medical aids were included in the analyses. For valuation, reference prices were used from the Dutch costing manual [19]. Costs for informal care were added to the healthcare costs and valued using the opportunity cost method [20]. Productivity costs were retrieved from self-reported data on absence from paid work and reduced efficiency at work and were calculated using the friction cost method [21]. Both healthcare utilization costs (including informal care) and productivity costs were estimated using two GLM models (one model for ST and one model for ERT), with age, gender, disease duration as explanatory variables (regression estimates are provided in Table S3 (see Additional file 1).

### Cost-effectiveness

Effects were expressed in life years gained and quality adjusted life years (QALYs) gained. QALYs are calculated as the number of life years gained corrected for the quality of life (i.e. utility) during these life years. Incremental cost-effectiveness ratios (ICERs) were presented as both incremental costs per life year gained and incremental costs per QALY gained. Probabilistic sensitivity analysis (PSA) was performed and the 95% confidence intervals (CI) were derived from the 2.5th and 97.5th percentiles of the PSA iterations.

### Model settings

A double-loop model was used to represent patient heterogeneity and parameter uncertainty [22]. The double-loop model consisted of 30 simulated populations of 90 bootstrapped patients (equal to the number of patients for which data for all patient characteristics that were used as input parameters in the cost-effectiveness model were available) and 1000 Monte Carlo simulations (see Additional file 1: Figure S1). The inner loop represented patient heterogeneity, the outer loop represented parameter uncertainty. Firstly, in the outer loop, values from the distributions of all regression coefficients in the models of survival, quality of life and cost models were drawn. These values were kept constant for a sample of 90 bootstrapped patients in the inner loop. Using the information on these 90 patients, individual estimates for survival, quality of life and costs were made, for both ST and ERT, and averaged over this population of 90 patients. This process was repeated 30 times in the inner loop. Then, this entire process was repeated 1000 times in the outer loop.

A lifetime time horizon was used in the base case analyses. Patients in the ERT (ST) group were modelled to receive ERT (ST) until death; i.e. in the model patients did not switch treatments. Effects were discounted using a discount rate of 1.5%; costs were discounted at 4.0%, as recommended by the Dutch pharmacoeconomic guidelines [23]. Discounting is done to adjust for time preferences; the further the gains and losses occur in the future the less weight they get.

The cost-effectiveness model was programmed in Microsoft Excel 2013 (Microsoft, 2013). Survival analyses were performed using R [24]. Regression models for quality of life and costs were estimated in Stata version 14.1 (StataCorp, 2015). The analyses were performed on patient level data until the year 2011.

### Sensitivity analyses

Two types of structural uncertainty were assessed by means of one-way sensitivity analyses. Firstly, a simpler regression model was used to estimate survival: survival was estimated using treatment as the only explanatory variable. Secondly, the influence of the time horizon on the ICER was assessed by using shorter time horizons for the cost-effectiveness analyses (i.e. time horizons of 5 years and 15 years).

Next to assessing the structural uncertainty of the model, the influence of specific input parameters on the outcomes was assessed in one-way sensitivity analyses. Price of medication was reduced by 20% to investigate the influence of the price of ERT on the ICER. Furthermore, analyses were run using a discount rate of 0% for both costs and effects. To test the influence of the utility gain on the ICER, utility gains were set to zero and to 0.1 in two separate sensitivity analyses. Similarly, increases in healthcare costs other than costs of ERT were set to zero to test for the influence of those costs on the ICER. Finally, a sensitivity analysis was performed in which the lifetable was used until year 8 (i.e. the point at which for at least 25% of the initial population data were still available for analysis), and this value was carried forward to later years. This sensitivity analyses was performed to assess the influence of the small sample size for the survival model in the ninth year.

## Results

### Patient population

Table 1 shows the baseline (i.e. first visit in the database) patient characteristics for the patients included in the cost-effectiveness model. The cost-effectiveness model included data of adult patients in the age range of 23 to 75 years at first visit.

**Table 1:** Baseline characteristics of Pompe patients included in the cost-effectiveness analyses (*n* = 90)

	Mean	Median	Range
Age at first visit	49.1 years	50.0 years	23.0–75.0 years
Disease duration (since diagnosis)	7.7 years	4.3 years	0.0–27.6 years
Female	48%		
Residual Enzyme activity (in fibroblasts)	12.0%	12.0%	0.5–19.9%
Wheelchair use	31%		
Ventilation use	27%		
Period at risk in ST survival analyses	3.5 years	3.5 years	0.0–8.9 years
Period at risk in ERT survival analyses	3.4 years	3.7 years	0.2–8.4 years

### Survival

**Fig. 1:**
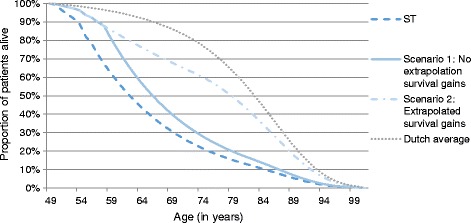
Survival curves for both treatments derived from the cost-effectiveness model and general population in the Netherlands (average patient)

Survival probabilities for ERT were higher than for ST (see Additional file 1: Table S1). The resulting survival curves for ST and ERT are given in Fig. 1. In both scenarios, survival increased substantially due to ERT. In scenario 1, a worse case scenario with no extrapolation of survival gains after the observed period, undiscounted life expectancy was approximately 2.6 years longer for ERT patients than for ST patients (i.e. the difference between the blue and dashed line), using a lifetime time horizon. Table 2 shows that incremental life years were 1.9 years when a 1.5% discount rate on effects was applied. Survival gains were even larger in scenario 2; life expectancy increased with 8.2 years without discounting, and 5.4 years when discounting was applied.

**Table 2:** Cost, effects and cost-effectiveness of ST and ERT (lifetime time horizon)

Scenario 1: No extrapolation of survival gains				
Incremental and total effects	ST	ERT	Difference	Difference 95% CI
Utilities	0.42	0.45	0.03	[0.02–0.05]
Life expectancy	16.33	18.21	1.89	[0.67–2.62]
QALYs	10.53	12.57	2.04	[1.30–2.57]
Incremental and total costs	ST	ERT	Difference	Difference 95% CI
Total costs	€ 329,105	€ 6,795,495	€ 6,466,827	[€ 5,686,402-€ 7,340,316]
Healthcare costs	€ 325,720	€ 6,790,671	€ 6,464,951	[€ 5,683,798-€ 7,342,186]
ERT costs	€ 0	€ 6,258,915	€ 6,258,915	[€ 5,513,466-€ 7,019,921]
Costs of drug administration	€ 0	€ 157,457	€ 157,457	[€ 95,399-€ 223,444]
Other healthcare costs	€ 325,720	€ 374,299	€ 49,390	[€ -264,511-€ 301,652]
Productivity costs	€ 3411	€ 5662	€ 2268	[€ -2916-€ 13,822]
Incremental cost-effectiveness ratios				
Cost / life year gained		€ 3,417,713		[€ 2,237,739-€ 10,714,797]
Cost / QALY gained		€ 3,167,914		[€ 2,348,946-€ 5,485,622]
Scenario 2: Extrapolated survival gains				
Incremental and total effects	ST	ERT	Difference	Difference 95% CI
Utilities	0.42	0.45	0.03	[0.02–0.05]
Life expectancy	16.42	21.84	5.44	[1.24–9.21]
QALYs	10.60	14.85	4.26	[1.77–6.62]
Incremental and total costs	ST	ERT	Difference	Difference 95% CI
Total costs	€ 324,967	€ 7,879,226	€ 7,554,844	[€ 6,885,851-€ 8,210,521]
Healthcare costs	€ 321,558	€ 7,874,627	€ 7,553,917	[€ 6,844,436-€ 8,210,008]
ERT costs	€ 0	€ 7,206,219	€ 7,206,219	[€ 6,684,091-€ 7,705,496]
Costs of drug administration	€ 0	€ 179,589	€ 179,859	[€ 106,760-€ 257,239]
Other healthcare costs	€ 321,558	€ 488,819	€ 168,109	[€ -172,810-€ 508,039]
Productivity costs	€ 3435	€ 5590	€ 2173	[€ -2684-€ 12,621]
Incremental cost-effectiveness ratios				
Cost / life year gained		€ 1,389,925		[€ 838,539-€ 5,317,415]
Cost / QALY gained		€ 1,774,390		[€ 1,164,826-€ 4,159,592]

Discount rate effects 1.5%; Discount rate costs 4.0%

### Quality of life

The average difference in discounted utilities found in the cost-effectiveness model was 0.03 (Table 2). Estimated utilities for both treatments decline over time, due to worsening clinical parameters which translate into utility decreases through the conceptual quality of life model. Discounted QALYs were 2.0 (scenario 1) and 4.3 (scenario 2) higher for ERT than for ST over a lifetime period.

### Costs and cost-effectiveness

Undiscounted annual treatment costs for an average weight patient was €450,000. Table 2 further shows costs for both treatments. In scenario 1, discounted lifetime incremental costs were approximately €6.5 million, consisting mainly (96.7%) of drug costs. Incremental costs for scenario 2 were €7.6 million, because patients lived longer and received ERT for a longer period of time. ERT did not reduce other healthcare costs, because ERT improves survival and during these additional years of life patients still need routine monitoring and other forms of healthcare and informal care.

For scenario 1, the ICER was estimated at €3.4 million per life year gained and €3.2 per incremental QALY. The ICERs for scenario 2 were lower; €1.4 million per life year gained and €1.8 million per incremental QALY.

**Fig. 2:**
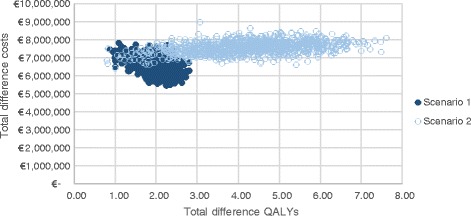
Cost-effectiveness plane; incremental costs and incremental effects of ERT over ST

**Fig. 3:**
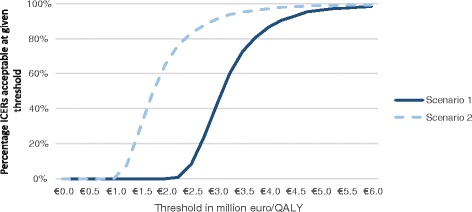
Cost-effectiveness acceptability curve: percentage of model iterations (y-axis) below CE threshold (X-axis)

A cost-effectiveness plane visualizes the variation in incremental effects and incremental costs, as it presents the results for each of the outer loop iterations. The cost-effectiveness plane in Fig. 2 shows the outcomes of the 1000 model iterations for both scenarios (i.e. each dot represents one outer loop, given the 30 simulated heterogeneous populations of 90 patients that were drawn in the inner loop). Uncertainty is primarily present concerning the difference in effects, especially when extrapolating survival gains in scenario 2. Uncertainty surrounding survival gains is the underlying determinant of the variation in PSA outcomes. The cost-effectiveness acceptability curve in Fig. 3 shows the percentage of simulations with a cost-effective outcome under a pre-specified cost-effectiveness threshold. Using a cost-effectiveness threshold of either €50,000 or €80,000/QALY (the upper limit in the Netherlands, depending on the severity of the disease), 0% of iterations would be considered cost-effective in either of the scenarios. When a threshold of €4.7 million per QALY is used 95% of iterations would be considered cost-effective in scenario 1, for scenario 2 this occurs at a threshold of €3.5 million per QALY.

### Sensitivity analyses

Table 3 shows the outcomes of the various sensitivity analyses. The effect of using a simpler model specification for survival, i.e. using treatment as the only covariate, on the ICER was limited. Changing the time horizon had the largest influence on the ICER. The ICER was higher with shorter time horizons; using a 5-year horizon the ICER was €15.6 million per life year gained (€7.0 million per QALY) and using a 15-year horizon the ICER was 3.9 million per life year gained (€3.6 million per QALY). Both incremental costs and effects were lower for the shorter time horizons than in the base case. The ICER increased mainly because of the lower survival gains. In the analyses with the 15-year time horizon discounted incremental life years were estimated to be 1.2 years. Discounted incremental life years were 0.1 years in the analyses with a 5-year time horizon; this survival gain was exclusively based on observed data. This is consistent with Fig. 1 and Table S1 (see Additional file 1), which showed that gains in survival increased after 5 years.

**Table 3:** Results one-way sensitivity analyses

	Incr. LY	Incr. QALY	Incr. Costs	Cost/LY	Cost/QALY
Scenario 1: No extrapolation of survival gains					
Base Case	2.03	2.13	€ 6,486,112	€ 3,195,040	€ 3,050,814
*Structural uncertainty*					
Survival: ERT as only covariate	2.15	2.08	€ 5,780,738	€ 2,692,018	€ 2,772,920
5 year time horizon	0.13	0.29	€ 2,043,440	€ 15,558,121	€ 6,958,412
15 year time horizon	1.19	1.31	€ 4,681,908	€ 3,944,770	€ 3,567,548
*Input values*					
Medication costs 20% reduced	2.03	2.13	€ 5,234,010	€ 2,578,258	€ 2,461,874
Discount rates 0%	2.61	2.69	€ 11,186,321	€ 4,287,545	€ 4,162,930
No utility gain	2.03	1.26	€ 6,486,112	€ 3,195,040	€ 5,138,186
Utility gain of 0.10	2.03	3.03	€ 6,486,112	€ 3,195,040	€ 2,139,947
No difference in healthcare costs except for cost of ERT	2.03	2.13	€ 6,418,842	€ 3,161,903	€ 3,019,173
Survival: last value carried forward from year 8 onwards	1.98	2.08	€ 6,387,051	€ 3,231,592	€ 3,073,535
Scenario 2: Extrapolated survival gains					
Base Case	5.67	4.38	€ 7,564,035	€ 1,334,081	€ 1,726,636
*Structural uncertainty*					
Survival: ERT as only covariate	5.44	4.14	€ 6,749,881	€ 1,241,847	€ 1,629,079
5 year time horizon	0.13	0.29	€ 2,043,440	€ 15,558,121	€ 6,958,412
15 year time horizon	1.59	1.58	€ 4,836,654	€ 3,047,385	€ 3,053,857
*Input values*					
Medication costs 20% reduced	5.67	4.38	€ 6,121,967	€ 1,079,741	€ 1,397,456
Discount rates 0%	8.22	6.12	€ 14,480,052	€ 1,761,281	€ 2,367,014
No utility gain	5.67	3.34	€ 7,564,035	€ 1,334,081	€ 2,265,117
Utility gain of 0.10	5.67	5.47	€ 7,564,035	€ 1,334,081	€ 1,382,324
No difference in healthcare costs except for cost of ERT	5.67	4.38	€ 7,390,957	€ 1,303,555	€ 1,687,127
Survival: last value carried forward from year 8 onwards	5,65	4.36	€ 7,478,037	€ 1,323,777	€ 1,716,538

Base case results differ somewhat from Table 2 as sensitivity analyses were performed using deterministic analyses

When an ERT-price reduction of 20% was modelled, incremental costs decreased to €5.2 million. The ICER decreased to €2.6 million per life year gained and €2.5 million per QALY gained. Without discounting, the ICER was €4.3 million per life year gained and €4.2 million per QALY. Discounting affected both costs and effects, but due to the differential discount rates it reduced the net present value of costs more than the net present value of effects. Disregarding utility gains reduced the incremental QALYs, but the impact on the ICER was limited. When the improvement in quality of life due to ERT was kept constant at 0.1 instead of 0.03 as derived from the quality of life model, incremental costs per QALY was €2.2 million. Excluding differences in other healthcare costs, or using survival data until year 8, had virtually no effect on the ICER. The sensitivity analyses for scenario 2 showed similar findings.

## Discussion

This study assessed the cost-effectiveness of ERT versus ST in adult patients with Pompe disease from a societal perspective. Survival increased considerably because of ERT. Using a lifetime time horizon the model showed that discounted life expectancy increased by up to 5.4 years when survival was extrapolated (scenario 2). Furthermore, ERT had a positive effect on quality of life of patients. However, the cost-effectiveness ratio is primarily determined by the costs of ERT. In the best scenario, this resulted in an incremental cost per life year gained of €1.4 million and an incremental cost per QALY ratio of €1.8 million.

The results of our analyses are in line with other studies that show that orphan drugs are usually not cost-effective under common cost-effectiveness thresholds, primarily because of their high prices. In comparison, ERT in Fabry disease for instance was associated with an incremental cost of €3.3 million per QALY gained [25].

When compared to various expensive cancer therapies, the effectiveness of ERT in Pompe disease is much larger in terms of absolute life years gained. For example, nivolumab increased survival of lung cancer patients by 0.61 years when compared to docetaxel [26] and pertuzumab increased survival of breast cancer patients by 1.4 years in comparison to trastuzumab/docetaxel [27]. This study has shown an increase in life expectancy due to ERT of 5.4 years (in scenario 2). Despite these larger effects, the cost-effectiveness ratio is less favourable for ERT in Pompe disease (nivolumab: €134,000 per QALY; pertuzumab: €148,824 per QALY) [26, 27]. As such, the study particularly shows the effect of the high price of ERT on the ICER. Medication costs of ERT are further increased because of the relatively high dose needed to reach muscle tissue; research has shown that a dosage of at least 20 mg/kg is needed to be effective [1, 11, 28].

In this study the list price of alguclosidase alfa was used. In March 2016, the market exclusivity period for alglucosidase alfa ended in Europe (and the manufacturer subsequently withdrew the product from the EU register of designated orphan medicinal products). This enabled other companies to enter the market with generic (cheaper) versions of the therapy. To date, no such alternatives exist.

### Strengths and limitations

Research in orphan drugs in general may be hampered by small patient numbers and lack of data and this also holds for cost-effectiveness studies [29, 30]. However, in the current study we had access to a large international longitudinal dataset with observational data to estimate survival. In addition, quality of life and costs were estimated by using an extensive dataset containing long-term follow-up on both ST and ERT. The availability of data for both ST and ERT was essential to perform an adequate cost-effectiveness study. When survival is affected, using an international dataset might be the only option to gather enough data to estimate cost-effectiveness. The availability of the relatively large amount of data for the various components in a cost-effectiveness study, i.e. survival, quality of life and costs, is exceptional, given the rarity of the disease.

Pharmacoeconomic guidelines prescribe the use of a time horizon that captures all benefits and costs of a treatment. In this respect, a lifetime horizon was most appropriate for modelling the cost-effectiveness in adult Pompe disease. If the time horizon is longer than the follow-up of the data (as was the case in this study), observed (survival) data need to be extrapolated. Because extrapolation (particularly of effects) is associated with uncertainty, especially when the time horizon is lifetime, we present two different scenarios. In scenario 1, in which we only include the survival gains in the observed period, we assume no gains in survival beyond the observed period, as a worse case scenario. In scenario 2, we extrapolated survival gains beyond the observed period. ICER estimates ranged from €3.2 million (scenario 1) to €1.8 million per QALY (scenario 2). Although this is a broad range in absolute terms, it also showed that the ICER is high even when the largest expected survival gain is modelled.

The number of patients in the dataset used to model survival was not sufficient to incorporate other explanatory variables in the Cox proportional hazard model than wheelchair use and ventilator support. Therefore, the effect of age on disease-specific survival could not be modelled. Hence, the effect of age on survival was limited to modelling of background mortality. A final limitation with regard to modelling survival was that beyond the observed period, the last-observed value from the life tables was carried forward. Actual survival was observed for a period up to nine years; estimated 9-year survival probabilities were extrapolated beyond this period. Sensitivity analyses showed that results were not affected when the values of year 8 were carried forward instead of the values for year 9. Ideally, a parametric survival function would have been fitted to the observed data, but this was not possible with the available dataset in which the same patients were on ST for a certain period after which they could switch to ERT. Information on long-term effects of ERT in Pompe disease with respect to survival is needed to assess the plausibility of the two scenarios.

The data used in this study were derived from observational studies. Observational studies can suffer from various types of biases, such as performance bias, selection bias and loss to follow-up [31, 32]. Performance bias occurs when patients and clinicians are not blinded, and can lead to an overestimation of treatment effects [32]. Selection bias can lead to differences in patient characteristics between groups, which can obscure the determination of treatment effects; i.e. when patients have different prognosis at start of treatment it cannot be established whether effects are caused by the treatment or by the initial differences in prognosis of patients [31]. Perhaps patients are in different stages of the disease when treated with ST or ERT. In our study, the impact of selection bias on the estimated survival was reduced because in the time-dependent Cox proportional hazard model, the patients that received ERT also contributed survival data to the ST group. Furthermore, by including age, gender and disease duration in the quality of life and costs regression models, and by using wheelchair and ventilator use in the survival model, we tried to correct for differences in patient characteristics as far as the data allowed. Loss to follow-up was limited for the Dutch patients in the study, because in the Netherlands all patients with Pompe disease are referred to the single expert centre at the Erasmus MC. Erasmus MC uses an extensive standardized follow-up protocol for all patients, which are either treated at the Erasmus MC, or elsewhere under supervision of Erasmus MC. Furthermore, loss to follow-up is reduced as patients are obligated to complete specific measurements and questionnaires. Hence, this study did capture the vast majority of the total Dutch population (>80%) of adult patients with Pompe disease.

### Policy implications

Evidence-based policy making and health technology assessment (HTA) may assist policy makers to effectively prioritise health interventions and make consistent decisions. This study showed that despite significant survival gains, the treatment for this very rare disease will never be titled cost-effective at the current price-level of the drug. Considerable price reductions will be needed to improve the cost-effectiveness ratio of this effective therapy. The exact causes of the high price for orphan drugs remain unclear, because a breakdown of this price in different components is not publicly disclosed. It could be due to several factors, such as high R&D costs, high production costs (particularly for complex manufacturing processes like the production of recombinant human alglucosidase alfa), lack of competition and the high perceived value of the drug. Transparency about price setting of orphan drugs is needed to justify their high prices, especially given the evidence on high gross profit margins on orphan drugs compared to other drugs [33, 34]. However, even if payers manage to negotiate price reductions, the European system of international reference pricing, in combination with parallel trade being legal, jeopardizes transparency about prices.

Unfavourable cost-effectiveness ratios do not only apply to the treatment of Pompe disease, but have been recognized for orphan drugs in general. To address this challenge, common collaboration between national healthcare authorities may support to increase negotiation power and reduce drug prices. Currently, the governments of the Netherlands, Belgium, Luxembourg and Austria collaborate in this respect [35], but this coalition preferably needs to be extended to other countries to improve the result of the negotiations. Transparency in price setting is a key issue in successful price negotiations, especially to safeguard payers from high prices for orphan drugs that result from misuse of orphan drug legislation (e.g. price increases for existing drugs after getting orphan designation; salami slicing) rather than (possible) acceptable reasons. It should be noted that treatment with alglucosidase alfa for Pompe disease is disease specific and cannot be used for other diseases. What could also contribute to reducing the ICER is to better target the therapy to those who benefit most. Start and stopping rules for ERT in Pompe disease have always been applied in the Netherlands and entail that treatment should only be initiated in symptomatic patients and treatment should be discontinued if patients do not show response to treatment. As more evidence becomes available, these start and stopping rules can be improved over time. European recommendations on these start and stopping rules have recently been published by the European Pompe Consortium [8, 36].

Whether the cost-effectiveness criterion should play a role in the reimbursement of orphan drugs has been debated, both in scientific literature [37–39] as well as in a broader societal setting [40]. Cost-effectiveness is used to maximize health under a given budget constraint. The cost-effectiveness threshold quantifies the societal willingness to pay for one unit of health gain. In the Netherlands, cost-effectiveness thresholds are used to guide discussions on cost-effectiveness, but these thresholds are not a conclusive reimbursement criterion. The Dutch threshold is dependent on the severity of a disease: for diseases with a severity between 0.1 and 0.4 the threshold is €20,000/QALY; for diseases with a severity between 0.41 and 0.7 the threshold is €50,000/QALY; and for diseases with a severity between 0.71 and 1 the threshold is €80,000/QALY [41]. The use of a higher cost-effectiveness threshold for orphan drugs has been suggested [42]. The basic questions are whether a societal preference for rarity and inherited diseases exists and how much society wants to avoid denying access to treatment for patients with these diseases. Several positive reimbursement decisions for orphan drugs and the existence of programmes designed specifically to give patients with rare diseases access to treatment in various countries (e.g. the Life Savings Drug Program in Australia; the Scottish Rare Conditions Medicines Fund) imply that policy makers believe this preference to exist. Empirical evidence on societal preferences for rarity is limited and mixed [43–46]. Further evidence is needed on societal preferences in other countries, including the Netherlands, and in what way policymakers incorporate these views in decision making. Theoretically, the cost-effectiveness threshold should reflect the opportunity costs of healthcare spending. When the ICER of an intervention is smaller than the threshold, the health gains of a new intervention exceed the health effects of the interventions that are displaced elsewhere in the healthcare system to compensate for the additional costs of the new technology. Empirical data on the value of displacement costs in the Netherlands are not yet available. Other criteria also seem to play a role in reimbursement decisions. A systematic literature review found nine other criteria that were used in decision making on orphan drugs: uniqueness of the indication, prevalence of the disease, disease severity, advancement of technology, complexity of manufacturing, unmet medical need, scientific evidence on effectiveness, drug safety, and budget impact [47]. In addition to these criteria, other studies identified the availability of alternative treatments, social impact of the disease and treatment, whether follow-up research will be performed, and whether the drug is disease modifying or not as criteria that can be important in reimbursement decisions on orphan drugs [48, 49]. From the perspective of the physicians and the patients, the fact that Pompe disease is still the first and only proven treatable inheritable skeletal muscle disorder may also play a role. The knowledge obtained may be used for the better understanding of similar diseases and in the development of next generation and other innovative therapies.

## Conclusions

This model-based cost-effectiveness study has shown the significant benefits of ERT in adult Pompe disease in terms of survival and QALYs over a life-time horizon. The study in this rare orphan disease could be performed due to the start of prospective collection of data 4 years before ERT was registered. It has also shown that the high price of ERT for this ultra-rare disease results in a cost-effectiveness ratio of ERT that by far does not meet the conventional threshold values.

## Additional file

Additional file 1: Table S1. Survival: Cox regression estimates. **Table S2.** Quality of life: regresssion estimates conceptual disease model. **Table S3.** Costs: regression estimates healthcare costs and productivity costs and input cost parameters**. Figure S1.** Double loop model structure. (PDF 394 kb)

## Abbreviations

CI: Confidence Interval
ERT: Enzyme Replacement Therapy
EU: European Union
GLM: Generalized Linear Model
HTA: Health Technology Assessment
ICER: Incremental Cost-effectiveness Ratio
IPA: International Pompe Association
PSA: Probabilistic Sensitivity Analyses
QALY: Quality Adjusted Life Year
ST: Supportive Treatment
