# Supplementary material for: Cost-effectiveness of enzyme replacement therapy with alglucosidase alfa in adult patients with Pompe disease
Source: Orphanet J Rare Dis. 2017 Dec 13;12:179. doi: 10.1186/s13023-017-0731-0 (PMC5729274; doi:10.1186/s13023-017-0731-0)
Supplement: Additional file 1: Table S1. — Survival: Cox regression estimates. Table S2. Quality of life: regresssion estimates conceptual disease model. Table S3. Costs: regression estimates healthcare costs and productivity costs and input cost parameters. Figure S1. Double loop model structure. (PDF 394 kb) [file 13023_2017_731_MOESM1_ESM.pdf]

Table S1 Survival: Cox regresssion estimates

**Time dependent Cox regression estimates ERT=0**					
		No ventilation		Ventilation	
	Year	Cumulative survival	Standard error	Cumulative survival	Standard error
No ambulation	0	1.000	0.000	1.000	0.000
	1	0.997	0.002	0.994	0.005
	2	0.985	0.006	0.965	0.015
	3	0.975	0.009	0.944	0.022
	4	0.965	0.012	0.921	0.029
	5	0.951	0.017	0.891	0.040
	6	0.919	0.027	0.825	0.063
	7	0.900	0.034	0.785	0.076
	8	0.871	0.045	0.728	0.094
	9	0.845	0.058	0.679	0.117
Ambulation	0	1.000	0.000	1.000	0.000
	1	0.992	0.006	0.982	0.013
	2	0.955	0.019	0.901	0.031
	3	0.928	0.027	0.843	0.039
	4	0.900	0.035	0.785	0.048
	5	0.862	0.046	0.712	0.061
	6	0.781	0.071	0.567	0.084
	7	0.733	0.086	0.490	0.095
	8	0.666	0.106	0.393	0.105
	9	0.609	0.127	0.321	0.122
**Time dependent Cox regression estimates ERT=1**					
		No ventilation		Ventilation	
	Year	Cumulative survival	Standard error	Cumulative survival	Standard error
No ambulation	0	1.000	0.000	1.000	0.000
	1	0.999	0.001	0.998	0.002
	2	0.995	0.003	0.989	0.007
	3	0.992	0.004	0.982	0.010
	4	0.989	0.006	0.974	0.013
	5	0.984	0.008	0.964	0.018
	6	0.973	0.011	0.940	0.026
	7	0.967	0.014	0.925	0.031
	8	0.956	0.017	0.903	0.039
	9	0.947	0.022	0.883	0.049
Ambulation	0	1.000	0.000	1.000	0.000
	1	0.998	0.002	0.994	0.005
	2	0.985	0.008	0.967	0.015
	3	0.976	0.012	0.947	0.023
	4	0.967	0.016	0.925	0.029
	5	0.953	0.021	0.897	0.036
	6	0.923	0.031	0.833	0.047
	7	0.905	0.004	0.795	0.052
	8	0.877	0.045	0.740	0.059
	9	0.852	0.056	0.693	0.075

Table S2 Quality of life: regresssion estimates conceptual disease model

Coefficients quality of life model						
	FVC	MRC	FSS	RHS	VAS	Utility
Constant	60.359*	63.185*	6.459*	24.887*	6.903	0.580*
Age	-0.089	0.130	0.020	-0.126*	0.084	0.000
Female	13.688*	-0.033	0.384	-1.551*	3.413	-0.011
Disease duration	-0.184	-0.257*	-0.067*	-0.035	0.512*	-0.006*
Enzyme activity	0.952	0.924*	0.061	-0.064	-0.073	-0.001
FVC			-0.008	0.079*		
MRC			-0.024*	0.120*		
FSS				-0.721*		
RHS					1.662*	
VAS						0.003*
ERT	0.118	1.370*	-0.045	-0.265	6.227*	0.028*
**Standard errors quality of life model**						
	FVC	MRC	FSS	RHS	VAS	Utility
Constant	10.421	5.052	1.128	2.917	10.294	0.097
Age	0.175	0.085	0.011	0.027	0.107	0.001
Female	5.256	2.437	0.302	0.735	2.589	0.036
Disease duration	0.202	0.102	0.017	0.043	0.152	0.002
Enzyme activity	0.672	0.318	0.043	0.105	0.375	0.005
FVC			0.006	0.014		
MRC			0.011	0.029		
FSS				0.135		
RHS					0.199	
VAS						0.000
ERT	0.559	0.341	0.100	0.253	1.169	0.011

Abbreviations: FVC = Forced Vital Capacity; MRC = Medical Research Council; FSS = Fatigue Severity Scale; RHS = Rotterdam Handicap Scale; VAS = Visual Analogue Scale; ERT = Enzyme Replacement Therapy

* Indicates significance at 0.05 level

Table S3 Costs: regression estimates healthcare costs and productivity costs and input cost parameters

**Regression estimates healthcare costs and productivity costs model ST**				
	Coefficients (log transformed)		Standard errors	
	Healthcare costs	Productivity costs	Healthcare costs	Productivity costs
Constant	7.958*	9.245	0.586	1.514
Age	0.031*	-0.046	0.011	0.027
Disease duration	0.021	-0.102*	0.016	0.035
Female	-0.525	-0.734*	0.275	0.572
**Regression estimates healthcare costs and productivity costs model ERT**				
	Coefficients (log transformed)		Standard errors	
	Healthcare costs	Productivity costs	Healthcare costs	Productivity costs
Constant	9.272*	10.921*	0.536	2.048
Age	0.002	-0.094*	0.009	0.037
Disease duration	0.045*	-0.050	0.011	0.026
Female	-0.457*	-0.213	0.222	0.683
**Treatment costs: regression estimates patient's weight**				
	Coefficients	Standard errors		
Constant	58.148*	2.592		
Age	0.493*	0.031		
Female	-13.360*	2.803		

**Treatment cost: input parameters**		
	Values	Remarks
ERT treatment costs / kilogram	€ 5,788	Dosage 20mg/kg; one infusion per two weeks
**Costs of administration**		
Costs of administration (per year)	€ 11,660	One infusion per two weeks
Home costs	€ 433	Source: bottom-up costing study (2014 prices)
Hospital costs	€ 507	Source: bottom-up costing study (2014 prices)
Proportion treated in hospital	21%	

* Indicates significance at 0.05 level

Figure S1 Double loop model structure

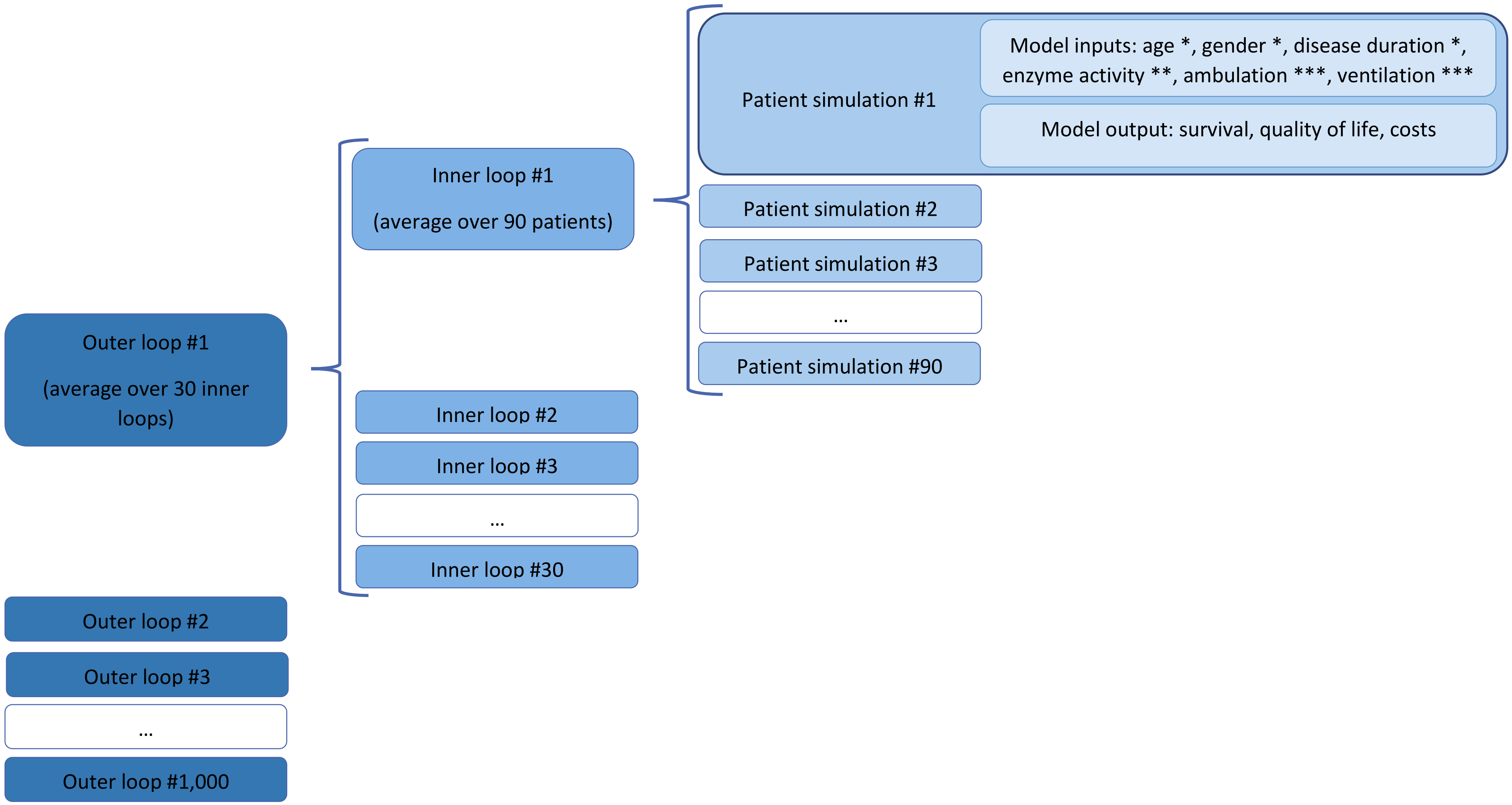

* Used as input variable for costs and quality of life models; ** Used as input variable for quality of life model; *** Used as input variable for survival model
